# On the Relationship between Economic Development, Environmental Integrity and Well-Being: The Point of View of Herdsmen in Northern China Grassland

**DOI:** 10.1371/journal.pone.0134786

**Published:** 2015-09-02

**Authors:** Xiaobin Dong, Guangshuo Dai, Sergio Ulgiati, Risu Na, Xinshi Zhang, Muyi Kang, Xuechao Wang

**Affiliations:** 1 State Key Laboratory of Earth Surface Processes and Resource Ecology, Beijing Normal University, Beijing, 100875, China; 2 College of Resources Science and Technology, Beijing Normal University, Beijing, 100875, China; 3 Department of Science and Technology, Parthenope University, Napoli, 80133,Italy; 4 Xilinguol Vocational College, Xilinhot, 026000, China; Chinese Academy of Sciences, CHINA

## Abstract

Inner Mongolia is among the most important regions in terms of contribution to the socio-economic development of China. Furthermore, its grassland is a major ecological barrier for Northern China. The present study evaluates the changes in ecosystem services availability and human wellbeing based on a survey on864 herdsmen of the grassland and 20 governmental officials. The survey provided the following results: (1) The supporting and provisioning services of the grassland have recently declined, thus affecting the herdsmen’s wellbeing. The intensity of grazing, coal exploitation and tourism development heavily limits the availability of ecosystem services, among which provisioning ones are perceived as the most important. Below a certain threshold, grazing and mining are likely to promote the improvement of herdsmen’s wellbeing, while trespassing that point, the enhancement of the herdsmen’s living standards is curbed. (2) The herdsmen’s cultural exchange, health conditions and social relations are better now than before; however, the threats on herdsmen’s health and safety have increased. (3) A better income is among the most urgent herdsmen’s needs. Livestock revenues did not increase at the same rate as the damage to the grassland and human risk exposure did. (4) The governmental ecological compensation policy did not entirely solve the problem of grassland desertification and environmental degradation, although it is also true that the degree of implementation and effectiveness of government policies is related to the still insufficient herdsmen’s understanding and acceptance of these policies.

## Introduction

Over the last thirty years, the complex relationship between ecosystem services, economic development and human well-being has been widely acknowledged, even if it has not been completely defined[1–5]. The degradation of ecosystem services often seriously affects human well-being, also leading to the loss of natural capital and national treasures [6].MEA [2,7]divides ecosystem services into provisioning, regulating, cultural and supporting services. It also emphasized that different ecosystem services contribute to different aspects of human well-being,which include basic needs, safety, health care, provision of education, rights, freedom and social relationships. Summers et al[8]divided human wellbeing into four main aspects, namely basic needs, economic needs, environmental needs and subjective happiness, and discussed the potential influences of ecosystem services on them. Their report highlights that piecemeal analytical techniques will likely undervalue these relationships and suggests the need for more holistic approaches to assess the role of ecosystem services in well-being increase.

Since the MEA results where published, several researchers pointed out the existence of trade-offs between ecosystem services, so that increases in one category of ecosystem services may lead to a decline in other categories due to socio-ecological dynamics[9–10], which creates winners and losers when ecosystem services change. Societal contexts and individual needs determine the way ecosystem services contribute to human well-being[11].Measures of well-being are primarily linked to social policies and economic status, while environmental factors are most often ignored in national accounts of human well-being despite the fact that the environment is vital to quality of life [12–19].Daw et al. [11]elaborated that aggregated analyses may neglect crucial poverty-alleviation mechanisms. Smith et al. [5]noted the need for integrating service flow values from natural, social, human and built capital to better understand the relationship between ecosystem services and human wellbeing and set up a predictive model consisting of economic services modules, a social services module and an ecosystem services module to evaluate human wellbeing.

As a big prairie country, China has several typologies of natural grassland over about 400 million hectares, accounting for 40% of land area. After the implementation of the “returning cultivated land to forests” policy, a “returning grazing land to grassland” policy was launched in 2003. In fact, due to natural causes and human factors such as overgrazing, 90% of the available natural grasslands showed varying degrees of degradation, leading to intensified desertification, drying up of rivers and lakes, dust storms and other natural disasters occurring more frequently. Grassland degradation is a widely observed problem and estimations for Inner Mongolia’s grasslands reported 30–50% to be degraded [20–22]. In this region, the environment is strongly affected by human activities, and the negative effects of the natural degradation phenomena are exacerbated. Hoffmann et al. (2008)[23]observed that in Xilinguole the grazing intensity was the most important factor affecting the material balance of land (nutrients and water input and output flows), and that its effects are reflected in the worsened vegetation conditions (vegetation height and coverage).Awareness of this problem increased in recent years ever since heavy sand storms originating from Inner Mongolia’s steppes hit Beijing in Spring more and more frequently[24].The grassland of Inner Mongolia is not only a source of production and life material for farmers’ and herdsmen’s survival and development, but is also an important green ecological barrier in northern China that keeps fresh water, maintains the carbon and nitrogen cycle, prevents desertification and contributes to the environmental integrity. Xilinguole grassland is located in the northeast of Beijing and not too far away from Bejing: a well preserved grassland can reduce soil erosion and prevent the desert spread by means of a natural line of defense; in this sense it plays the role of an ecological barrier. The Xilinguole grassland is also an ideal place for recreation, tourism and scientific research[25].

The grassland ecosystem provides a variety of service functions, among which climate regulation, water and soil conservation, conservation of biodiversity, nutrient cycling, leisure and entertainment[26–27]. An appropriate evaluation of direct and indirect grassland ecosystem services could help people understand their full value for wellbeing and economic sustainability and enhance the awareness of needed ecosystem protection.

Actually,in the past centuries, animal grazing was the main activity for herdsmen to gain an income.To some extent, more animal grazing means more income, but of course more grazing activity means more ecological pressure on the grassland, thus reducing thegrassland ability to provide direct and indirect ecosystem services.After thousands of years of nomadic animal husbandry activities, northern China temperate grassland shows widespread degradation, followed in the past half century by increased grain production in the 1970s, overgrazing since the 1980s, global warming and prairie drought since the 1990s, and intensified coal mining in the 21st century. State Statistics Bureau point out that livestock overloading rate in the grassland of the Inner Mongoliagot to 116.7% in 2012.If grazing activity were stopped, the grassland would grow much better in so increasing itsresilience, wild animals would come back and finally the hierarchy of the ecosystem food chain would be restored and would allow therecovery of ecosystem service functions

The change in ecosystem services and human wellbeing has an effect on the landscape. Landscape sustainability science enhances the understanding of and improves the dynamic relationship between the ecosystem and human well-being in a changing landscape. Spatially explicit methods and experimental approaches are essential to coupling the dynamics of ecosystem services and human well-being[28].Considering the significance and vulnerability of the Xilinguole grassland, deeper and more comprehensive empirical studies are required to identify an explicit link between the influence of human activities, the ecosystem services of the grassland in Inner Mongolia and human well-being. Currently, most studies mainly proceed from the perspective of human needs satisfaction and do not systematically and comprehensively address the interplay of human well-being and ecosystem integrity. In-depth understandingof how changes in the ecosystem services in Inner Mongolia affect human well-being and what measures the state government should take to improve the ecosystem service level and maintain human well-being are important issues that are addressed in this study. In particular, the responses of the herdsmen are the starting point for a better understanding of what they perceive as well-being and lifestyle improvement, and what they aim to achieve as a follow up of governmental policies. This research attempts to evaluate the changes in ecosystem services and human well-being based on a survey of 864 grassland herdsmen and 20 governmental officials, in order to facilitate resource conservation and ecological compensation towards sustainable development.

## Materials and Methods

### 2.1 Study site

**Fig 1:**
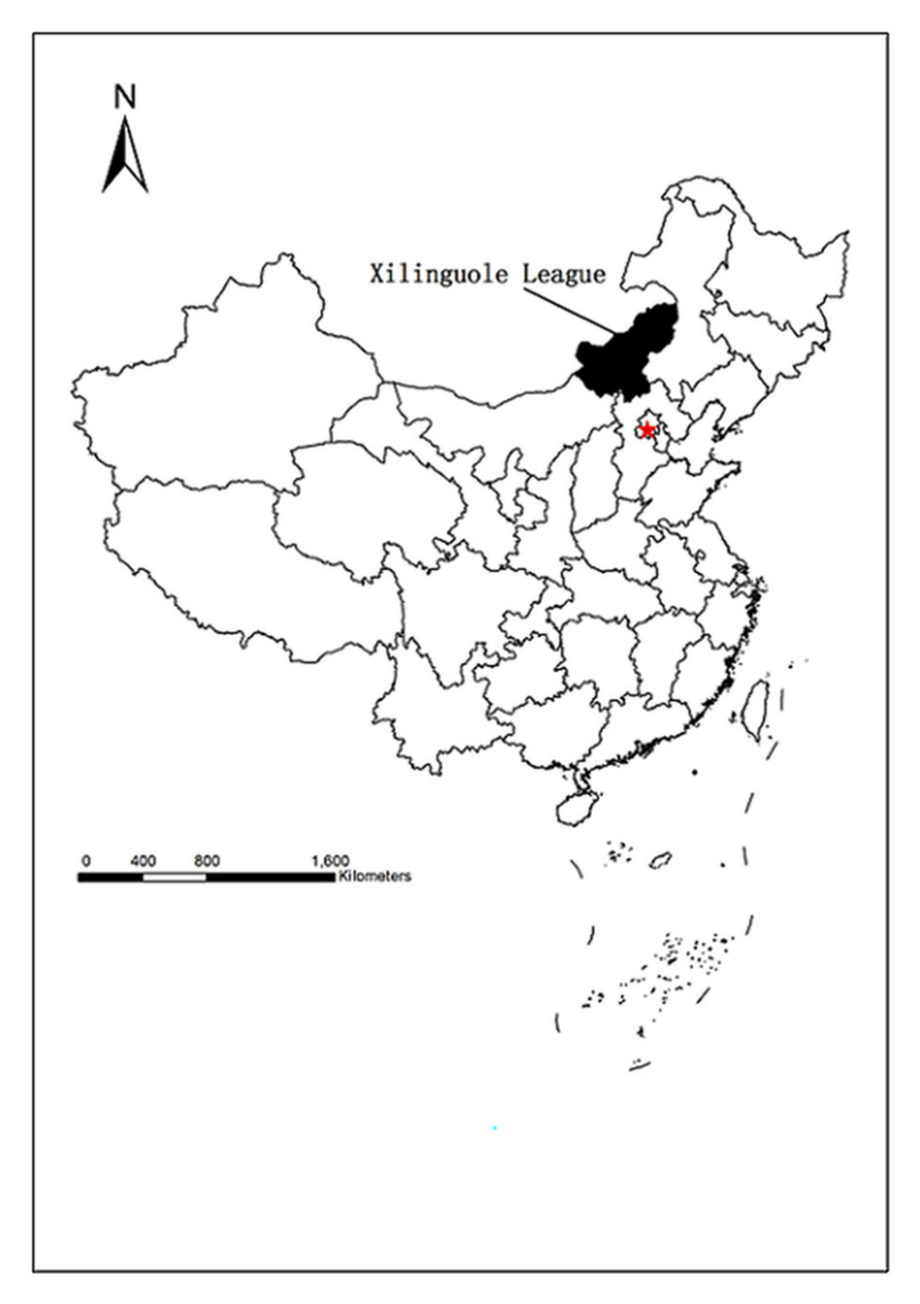
Xilinguole League (highlighted in black) China.

This research was carried out in Xilinguole League, which lies in the middle part of the Inner Mongolia Autonomous region, in northern China, ranging from East longitude115° 25' to 119° 58'and North latitude from 41° 35' to 46° 46' (Fig 1)[29]. It covers an area of 19,988,400 ha, 89.9% of which is high-quality natural grassland[30]. Xilinguole experiences dry continental monsoons with an annual average temperature of 0°C. Average annual rainfall generally ranges between 400 mm in the eastern part to 150 mm in the western part. Most rain occurs in July, August and September.

As one of the four major grasslands around the world, Xilinguole is rich with natural resources and species diversity. The national Grassland Nature Reserve of China was brought into the international monitoring system by UNESCO. The region also is the most important livestock product base in China. The number of head of livestock reached 12,881,900 in the year 2013, an increase of 9.3% over the previous year; the total livestock increase was 1,096,600 units, with a growth rate of 5.3% compared to total livestockin 2010. The herdsman’s production style has changed in the last decades. Now most of the herdsman use allocated grasslands. The herdsmen who keep the traditional nomadic translocation are less than 5%. In winter most of the herdsman feed their animals in permanent barns with dry grass that they harvest in summer.

The total population of Xilinguole League was 1,002,600 persons in 2010 [30]. The economy of this area mainly depends on animal husbandry, primary commodities and tourism. The average herdsmen income was 10,109 Yuan RMB in the year 2013, an increase of 13.3% compared to 2012. Instead, the average income of other citizens was around 22,708 Yuan RMB, 10.7% higher than in the year 2012. Grazing land is passed down through families, but not privately owned. With respect to the State-owned grasslands, the State Council shall exercise the right of such ownership on behalf of the State, and the herdsman has the right to use grasslands with responsibility system of management under contract.

The region includes 13 natural reserves, out of which two National Nature Reserves and seven regional level protected areas. The two national protected areas cover a surface of 680,000 hectares, while the regional nature reserves cover an area of 970,000 ha. Environmental quality is monitored by 13 environmental monitoring stations. Also due to these protected areas, the number of tourists in the grassland increased to 11,106,100 in 2013,13% more than in 2012. A number of environmental protection policies were implemented, such as:combination of protection and construction, with special attention on protection aspects; returning cultivated land to forests and returning grazing land to grassland; pollution control at the source.

The Xilinguole area is also rich with mineral resources, with more than 80 types of minerals extracted. Coal resources are extremely abundant. According to coal exploration data, the Xilinguole League has a total of 188.3 billion tons of coal reserves and contains the second largest reserve in Inner Mongolia [31].Coal production was 1.4 million tons in 2013, while it was 1.1 million tons in2010. There are now 25 big mines in the region, less than in the past, because several small companies merged into big companies.

However, increasing large-scale human activities (such as coal exploitation, over-grazing and so on)in the grassland are seriously affecting the local environment, in so also affecting the well-being of local populations. Understanding the potential impacts of present and future growth on both human wellbeing and natural systems is a challenging and timely topic to the local governments and herdsmen.

### 2.2 Method

We did not seek ethics approval in this case because the questionnaire did not involve any personal privacy or any ethical questions, or any animal experiment, we only need the views of herdsmen about changes in ecosystem services and human well-being, but we approached our research institution's ethics committee prior to the beginning of this study for approval and got approvement. We informed consent from the caretakers (students’ teachers) on behalf of the minors/children enrolled in my study. The consent was verbal since the aim of questionnaire was told by their teachers, and teachers sent the questionnaire to those students who were interested in attending and answering the questionnaire.

A questionnaire (see Appendix for full text) was designed to investigate how responses from herdsmen and government officials about recent changes in the ecosystem services and human well-being were related to demographic and lifestyle factors (the respondent’s age, education, net income per year, location and other features). In September of 2011, four towns (Abaga, East Ujimuqin, West Ujimuqin and Sunide Left) in Xilinguole League were selected as study areas. Questionnaires were distributed and explained in one college and one middle school in Xilinguole city, asking the students to bring them home and help parents to fill them out. In so doing, problems with correct understanding and writing in Chinese were overcome, thanks to the students support and explanations. Of course, no instructions about the content and the values at stake were provided, not to alter the results of the survey. After one month, questionnaires were collected and processed. The involvement of students also increased the probability that the answers reflected the point of view of the entire family. According to Landeghem’s research[32], human wellbeing is different and differently perceived at different ages. Therefore, the herdsmen were divided into five age groups (20–29, 30–39, 40–49, 50–59 and beyond 60) that formed a total sample size of 1000 for this survey. After excluding erroneous responses (e.g., the selection of more than one response to a question or the omission of one or more responses), we obtained 864 satisfactory questionnaires (i.e., n = 864 valid interviewees).Moreover, 20 government officials were also interviewed.

The questionnaires were designed to ascertain the views of herdsmen about changes in ecosystem services and human wellbeing and how these changes have affected their behavior. Our survey includes data on the changing grassland ecosystem services, the respondent’s income, material needs for basic living, health condition, cultural exchange, education, freedom and choices rights. Our analysis integrates and compares data from the questionnaire and from Xilinguole League Statistical Yearbook [30].

## Results

### 3.1 The value of the ecosystem services of Xilinguole League grassland

The evaluation of grassland ecosystem services could help people understand the value of these services and enhance the awareness of ecosystem protection.The main goal is making people realize that the direct and indirect ecosystem services of the grassland supply valuable base in maintaining human well-being.

According to the degree of degradation, the grassland is divided into segments of grassland without degradation, or mild, moderate and severe degradation. Out of a total grassland area of 17.96×10^6^ha, the area without degradationis 10.27×10^6^ha, while areas presenting mild, moderate and severe degradation are respectively 3.68×10^6^ha, 1.93×10^6^ha and 2.08×10^6^ha[33].These data weregraphically calculated from remote sensing maps[34], according to percentages of each typology. The primary production of grassland ecosystem in Xilinguole League is1.8 x 10^7^t/yr based on the consideration of the degree of the degradation of this ecosystem[35, 36], while the gross biomass is 1.0×10^7^t/yr(Table 1).

**Table 1:** Biomass production of steppe ecosystem in Xilinguole League.

Type of grassland	Area (10^6^ha)	Biomass (10^4^t/yr)	Productivity (10^4^t/yr)	Percentage of area (%)
Grassland without degradation	10.3	746.7	1276.9	70.2
Mild degradation Moderate degradation Severe degradation	3.7	218.6	373.7	20.6
	1.9	60.4	122.2	6.7
	2.1	26.8	45.9	2.5
**Total**	**18.0**	**1052.5**	**1818.7**	**100**

Degradation means loss of fractions of natural capital and related ecosystem services. Dong et al.[37] recently performed an emergy-based assessment of the value of selected ecosystem services in the Xilinguole region(Table 2). Emergy values were converted to emergy-based currency equivalents (virtual monetary value, em$, indicating the potential contribution of these resource flows to the economy), translating into: provisioning services, 2.86 billion em$/yr; regulating services, 3.43 billion em$/yr; supporting services, 1.67 billion em$/yr; cultural services, 2.28 billion em$/yr, totaling 10.24 billion em$/yr that is about 3 times the actual monetary value estimated based on market dynamics (3.50 billion $/yr).

**Table 2:** Comparison of the environmental (emergy) and monetary values of selected activities related to the environmental services of the Xilinguole League grassland. (*)

Note	Activity	Emergy value	Currency Equivalents	Market value
		(E+20 sej/yr)	(E+07 em$/yr)	(E+07 $/yr)
Provisioning services				
1	Livestock products	147.16	253.72	63.86
2	Wind electricity	18.70	32.24	20.62
Regulating services				
3	Carbon sink	98.30	200.62	130.98
4	Water cycling	82.44	142.14	no market value
Supporting services				
5	Net primary productivity (NPP)	98.30	167.18	3.38
Cultural services				
6	Organized recreation	132.39	228.26	131.29

(*) Calculation details and from the Annex of Table 5 (Dong X.B, Yu B.H., Brown M.T., Zhang X.S., Ulgiati S. Environmental and economic consequences of the overexploitation of natural capital and ecosystem services in Xilinguole League, China. Energy Policy, 2014,67:767–780).

These results suggest that ecosystem services value is underestimated by markets and therefore policies aimed at implementing them may lead to increased development and wellbeing.

### 3.2 Herdsmen’s response to changing ecosystem services

Based on official statistical data[30]the number of people working in the livestock sector of Xilinguole grassland increased steadily in the last 10 years:from 108,091 people in the year 2000, to 124,777 people in the year 2005 and finally 130,944 in the year 2010. As a consequence, the livestock sector can be considered vital and important in the economic system of Xilinguole League and requires special attention.

Human well-being includes many factors that differ from person to person. We developed a core set of well-being domains representing relevant common themes across various measures in combination with the MEA and the local situation in the Xilinguole grassland.Income, basic needs, safety, health, education, social relationships and freedom and choice rights were selected to reflect human well-being.

**Fig 2:**
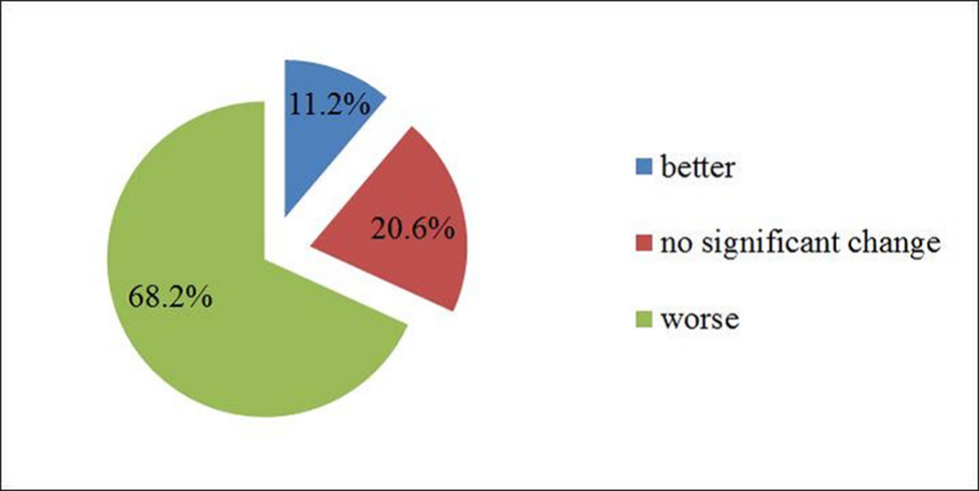
How has the grass grown in recent year?

When asked about grass growth in recent years, 68.2% of the surveyed herdsmen deemed that the grass growth was worse than before, 20.6% and 11.2% of the respondents answered no significant change and better than before, respectively. These replies, coupled to data from Table 1 indicating 30% of land showing moderate or severe degradation, suggest that the grassland growth conditions are getting worse, which directly affects forage production and reflects the decline in the ability to feed livestock, an important aspect of the declining grassland’s provisioning service (Fig 2).The herdsmen perception is confirmed by Wang et al.[38], who proved that overgrazing had decreased biodiversity in grassland.

**Fig 3:**
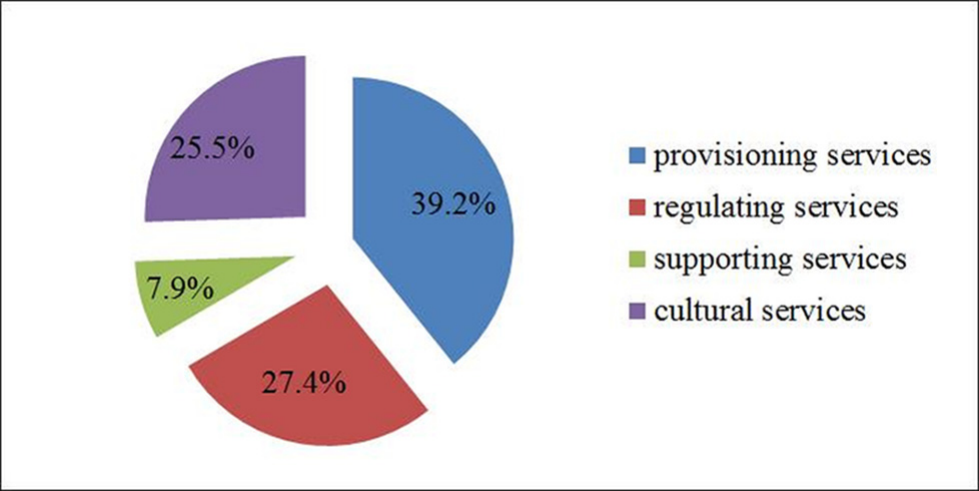
In the herdsman's view, which grassland ecosystem services are the most important?

When asked which of the grassland ecosystem services were the most important among a set of possible alternatives suggested to herdsmen(1.provisioning service; 2. regulating service; 3. culture services and 4. supporting services),responses were as follows:39.2% of surveyed herdsmen indicated provisioning services, 27.4% thought regulating services were the most important function, 25.5% of herdsmen suggested cultural services, while only 7.9% of respondents thought supporting service played the most important role(Fig 3). These data indicate that herdsmen paid more attention to the grassland provisioning services that maintain their sustainable livelihoods than to other services. However, 27% of herdsmen selected regulating services as the most important service function of the grassland, which indicates they also payattention to the grassland’s ecological environmental protection function.

**Fig 4:**
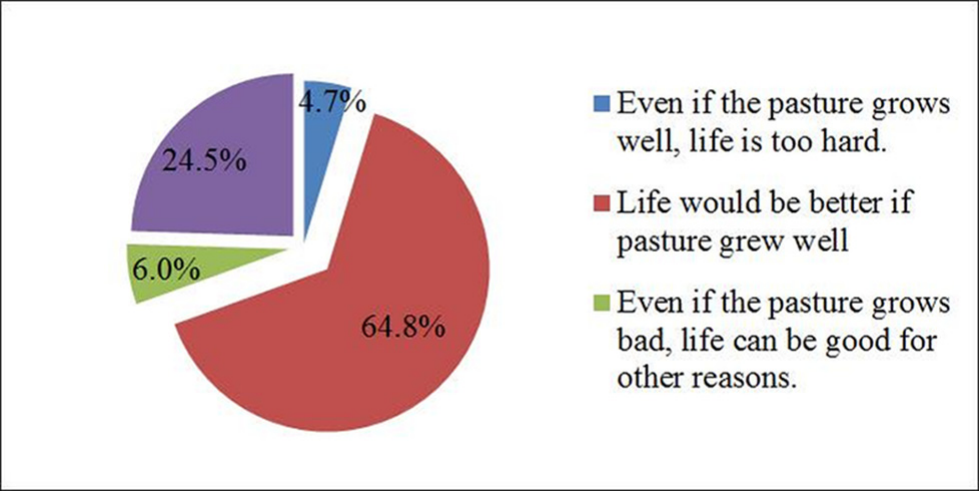
What is the relationship between pasture growth and herdsman life?

When asked “What is the relationship between pasture growth and herdsman life?”, 64.8% of the surveyed herdsmen said that their livelihoods would be better if the pasture grew well. Clearly,herdsmen still rely on grazing in their hope for improved living standards (Fig 4).Respondents seem still unable to escape the traditional and extensive mode that an increase of the number of livestock will improve their income and life standard.

#### 3.2.1 Income

Income perspectives play an important role as a mechanism to contribute to poverty alleviation[11]. Increasing income in pastoral areas serves as a means to improve well-being through the ability to purchase goods, access healthcare, allow tourism and create educational opportunities for children.

Regarding the net annual income of the herdsmen, 47.3% reported that their household annual income was between 10 thousand RMB(about 1588$)and 30 thousand RMB(about 4762$). One quarter (23.6%)responded that their family annual income was over 30 thousand RMB(about 4762$).Finally, 29.1% of herdsmen reported that their household annual income was still less than 10 thousand RMB(about 1588$) (Table 3). Results indicate that the population with a household annual income between 10 thousand and 30 thousand is the largest. The difference is not so significant when compared with the household net annual income of Xilinguole, being the herdsmen’s income very close to the average level. Only 21% of herdsman reported that their income increased, 43.0% of the herdsmen thought their income changed slightly and 35.6% of herdsmen reported that their income decreased.The largest fraction of herdsmen indicated that their income had not changed significantly. The main reasons for increased income were an increased amount of livestock and part-time work. In order to increase their income,45% of herdsmen have another job in addition to caring for their livestock, with 54.9% relying on grazing. Additional part-time jobs are considered a solution by 40.8%of herdsmen, while 39.9% of herdsmen holding a small grassland areas believe that a potential benefit may come renting more land; 19.3%of herdsmen look for coal mining or small businesses. Herdsmen are still linked to the traditional view that increasing the amount of livestock will improve their income.However, the biggest problem of this option is that it is seriously affected by natural conditions, with a low ability to withstand a variety of risks. To achieve sustainable herdsmen well-being, their income structure should not depend on only one source of income but should rather be stabilized through increasing the proportion of income fromother sources (e.g., secondary and tertiary industries).

**Table 3:** The income state of herdsmen.

**How much is the net annual income of your family?**				
Less than 10 thousand RMB	Between 10 and 30 thousand RMB		More than 30 thousand RMB	
29.1%	47.3%		23.6%	
**How has the net annual income of herdsmen changed?**				
Increased	Small changes		Decreased	
21%	43. 0%		35.6%	
**What caused the income changes?**				
The amount of livestock.	The total yield of grain.	The time of work outside.	Income from visitors.	Other ways
49.8%	10.7%	20.6%	6.5%	12.4%
**Do you have any other income resources besides selling livestock and grain?**				
Yes	No			
45.1%	54.9%			
**If the area of grassland is not enough for your needs, what measures should you take?**				
Find part-time work	Rent more grassland area		Other	
40.8%	39.9%		19.3%	

#### 3.2.2 Basic material needs

At present, only 20.1% of herdsmen reported that they are better-off; 67.1%of the herdsmen thought they had enough food and clothing. However, 12.3%reported that they live in poverty. Thus, more than 85.0% of the herdsmen perceive that they have gotten out of poverty (Table 4).

**Table 4:** The basic needs situation of herdsmen.

**Which level represents your living situation?**				
Needy	Have enough to eat and wear			Well-off
12.8%	67.1%			20.1%
**Is there electricity in your house? If there is, how do you obtain it?**				
Not yet	Yes, from coal-fired power plants			Yes, from wind power plants
1.4%	77.0%			21.6%
**Is the area of grassland enough for your grazing needs?**				
Does not meet their needs	Barely meets their needs			Fully meets their needs
49.0%	35.7%			15.3%
**What is the grassland area per person?**				
Less than 66.7 ha	Between 66.7 ha and 133.4 ha			More than 133.4 ha
25.9%	60.0%			14.1%
**What vehicle does your family own?**				
Motorcycle	Bicycle	Car	Truck	Carriage
49.8%	10.8%	20.6%	6.5%	12.3%
**What is your house made of?**				
Thatched cottage	Brick house			Yurt
4.2%	90.6%			5.2%

A number of basic material and energy needs are indispensable components of herdsmen daily life. Electricity is one of these. With the development and establishment of coal-fired and wind power plants, 98.6% of the herdsmen reported that they could obtain electricity. In this aspect, herdsmen’swell-being has dramatically improved. At present, 21.7% of herdsmen use the electricity from wind power(Table 4). Wind energy is cleaner than coal, and its wider application may reduce the usage of non-renewable resources such as coal and decrease CO_2_ emissions.It also reduces herdsmen’s electricity expenditures and the environmental damage brought about by coal mining in grassland and thus improves the herdsmen’s well-being.

Among the interviewed herdsmen, 49.0% complained that their grassland area is too small to meet their needs for grazing, 35.7% of the herdsmen’s pasture can barely meet their needs, and 15.3% of herdsmen were satisfied with their grassland area. This reflects that herdsmen still aim to obtain more grassland area to increase their livestock breeding proportion and add more income. The per capita area of one quarter (25.9%) of investigated herdsmen’s was less than 66.7 ha, while14.1% reported more than 133.4 ha, and most of them (60.0%) reported a per capita area between 66.7 ha and 133.4 ha.

At present, the main vehicles herdsmen use are cars, trucks, motorcars, bikes and carriages.Among these, motorcar use accounted for 49.8%, and the proportion of cars is relatively high at 20.6% (Table 4).The proportions of trucks and carriages are smaller;the fact that cars and motorcars have gradually replaced bicycles and carriages shows that herdsmen’s transport methods have been greatly improved. Herdsmen are more dependent on modern transport, which not only reflects the fact that communication between herdsmen and the rest of the world is improving, but also that their well-being is increasing.

Currently, 90.6% of surveyed herdsmen settle in houses made of brick and cement, replacing the yurt.The change in dwellings has been a gigantic transformation of the herdsmen’s life-style(Table 4), which also reflects their desire to pursue a more modern life-style by accepting the new dwelling style. The sturdiness and ability to resisting snow and wind are the most important reasons that herdsmen prefer to live in brick houses.

#### 3.2.3 Health

The domain of health includes expenditures for personal health, life expectancy and mortality, and physical and mental health conditions [5]. In addition, people’s health is related to lifestyle, behavior and healthcare. Food and drinkable water are aspects that affect human health, and the quality of the living environment is an external factor that influences physical and mental health.

The life expectancy of Xilinguole League is 71.3 years.This expectancy is inferior than the 77.9 years of the average Chinese life[39].In this area, most herdsmen report that their expenditures in medical treatment were below 5,000 RMB per year;only 7.9% herdsman spent money beyond 10,000 RMB in a year to see a doctor (Table 5).The fees for doctors are much higher than before, partly because the price of medical treatments increased but mainly due to herdsmen paying more attention to health and assigning more importance to physical examinations. Among the investigated herdsman, 58.6% of the herdsman’s villages have a clinic that solves basic medical problems. At present, the coverage of the rural cooperative medical system is over 80.0%(Table 5).This system helps herdsman decrease their expenditures in medical treatment and alleviates their economic burden. Health is an important component of herdsmen’s well-being.The establishment of clinics and popularization of the rural cooperation medical system reflects the government’s efforts to improve human health conditions and levels.

**Table 5:** Health conditions,medical treatment and public health of herdsmen.

**How much money did herdsman spend on seeing a doctor (in RMB per household per year)?**			
Less than 1,000 RMB	Between 1,000and 5,000 RMB	Between 5,000 and 10,000RMB	More than 10,000RMB
32.5%	41.7%	17.9%	7.9%
**Is there a clinic in your village?**			
Yes		No	
58.6%		41.4%	
**Does the village carry an endowment insurance system?**			
Yes		No	
81.8%		18.2%	
**Are you satisfied with the endowment insurance system?**			
Yes		No	
78.1%		21.9%	

#### 3.2.4 Safety

The index of safety in this area is frequently evaluated considering resource safety, property and life safety. Resource safety mainly means pasture and water quality and availability for livestock. Property and life safety involves the amount of livestock, the number of snowstorms that occur and violent crime and property crime rates.

Overall, the trend in outbreaks of grassland pests has gotten worse, largely due to drought in recent years, but not excluding the possibility of an effect of grassland degradation caused by overgrazing. Grassland pests and diseases are a direct threat to pasture forage production and indirectly affect the development of the livestock, reduce the income of the herdsmen and pull down their well-being. Controlling grassland pests requires engineering and biological measures, such as building grassland food chains. When asked about the occurrence of locusts, pests and diseases in the grassland, 47.2% of the herdsmen thought that they were more serious than before, 36.7% reported that the situation had changed little, while 16.1% of the herdsman thought this phenomenon was not serious(Table 6).

**Table 6:** Herdsmen perception of safety in Xilinguole League.

**How have locusts and other diseases changed in the grassland?**			
Aggravate	No change	Weaken	
47.2%	36.7%	16.1%	
**Is there enough water for your daily life?**			
Yes, it’s abundant.	Just to meet the needs.	No, there is water shortage	
42.3%	44.3%	13.4%	
**How do snowstorms affect people’s life?**			
Heavily affect herdsmen’s normal life	Small effect, people can live normally	No effect	
55.8%	41.7%	2.5%	
**Does the snowstorm threat your life safety?**			
Yes, we feel unsafe.	No, we can control that.		
52.4%	47.6%		
**What measures have you taken against snowstorms?**			
Purchase or prepare enough forage	Find a part-time job	Ask help from government	Take no measures
62.5%	10.4%	22.7%	4.4%

Xilinguole League lies in a semiarid region; therefore,fresh water is notably important for local development. Xilinguole League provides 310 million cubic meters of water per year for industrial uses, in addition to supplying drinkable, ecological, and agricultural water with a guaranteed rate of 97%. According to Wang et al[40], one ton of coal exploitation requires approximately 2.5 tons of water resources. In 2010, about 108 million tons of coal were mined in the Xilinguole League, which means that 267 million tons of water were used. This water consumption is so enormous that it seriously affects other industries’ water utilization and the underground water level as well. Moreover, soil erosion decreases the soil water holding ability and therefore increases the loss of fresh water.Water consumption will steadily increase with the production of coal, until the water resources available in the Xilinguole League will be no longer sufficient. Coal mining utilizes 86% of the annual water consumption and reduces the water available for herders’ daily life and production, threatening the basic living needs of the herders. The available water resources continue to decline as a result of excessive use of both surface water and ground water. Even worse, the water was polluted by the industrial sewage, threatening human life.

When asked whether there was enough water for daily life, 42.3% of the responses were positive, and 44.3% of surveyed herdsmen thought that the available water just meet their needs.The remaining 13.4% of responses reported that they lacked of fresh water in their daily life, and some areas have had water shortages(Table 6). However, the quality of the water is alarming; most drinking water of pasturing areas is below hygienic standards[41].Water is the source of life, so water scarcity will negatively affect people’s lives. Solving the water problem is serious issue in improving herdsmen’s well-being.

Snowstorms are one of the most important disasters in the grassland.If the livestock die in a snowstorm, it will have tremendous effect on herdsmen’s income. The results of the survey showed that over 55.8% herdsman deemed that blizzards influenced their livestock safety, as it destroyed herdsmen’s houses and barns and caused the loss of livestock, reducing herdsmen’s income. Over half (52.4%) of the herdsmen thought that snowstorms were a serious threat to their life safety and decreased their well-being. During the survey, 62.5% herdsman said that they built stronger barns and prepared enough forage for livestock to eat in the winter, while 22.7% herdsman had to ask for help from the government when snowstorms came. Herdsmen are highly dependent on forage, and snowstorm threatens human safety and their income, decreasing the level of human well-being(Table 6). The increase of snowstorm and drought enhance the survival risk, in that they affect the livestock, which is the main source of support and income to the herdsmen.

#### 3.2.5 Education

The culture and education of herdsmen includes the infrastructures and availability of schools, the education that their children receive and the time herdsmen are able to invest to participate in cultural activities.

When asked at what age children start going to school, 90.2% of responses reported that their children go to school at the legal age, although some go to school beyond 8yearsold. At present, most people graduate from middle school and high school.Fewer people drop out of primary school than in the past, which shows that herdsmen consider children’s education important and also indicates that the compulsory education system is more effectively carried out in this area (Table 7). However, there are not enough local schools, so the children live relatively far away from the school, and that is not convenient. The survey found that 52.9% of the respondents live 5 km away from the nearest primary school, 53.4% live 10 km away from their middle school, and the farthest distance is nearly 240 km, which causes severe problems for young students. Many resident students only go back home once or twice a month because travel is not convenient in pasture areas. Although the schools are far away from home, the quality of facilities and infrastructures are perceived as very good. A total of 94.2% of herdsmen are happy with the facilities in the schools, and 95.7% of them give good assessments of the teachers (Table 7).

**Table 7:** Education of herdsmen’s children.

**When do the children start going to school?**		
Before 8 year old		After 8 year old
90.2%		9.7%
**How far is the nearest primary school from home?**		
1–3 km	3–5 km	Beyond5 km
8.5%	38.6%	52.9%
**How far is the nearest middle school from home?**		
Less 5 km	5–10 km	Beyond 10 km
10.1%	36.5%	53.4%
**How are school facilities?**		
Very good	Sufficient	Not good
44.9%	49.1%	5.8%
**How about the teaching level of school?**		
Very good	Sufficient	Not good
59.7%	36.4%	3.9%

Although some problems still need to be addressed, the school directors try their best to build a good atmosphere for students, which improves the education conditions of the herdsmen and provides a critical foundation for the improvement of herdsmen’s well-being. Educational progress promotes other wellbeing domains, such as individual economic returns, achievements and accomplishments, enhanced worker productivity, lower crime rates and greater civic participation [42–43].

#### 3.2.6 Social relationships

**Fig 5:**
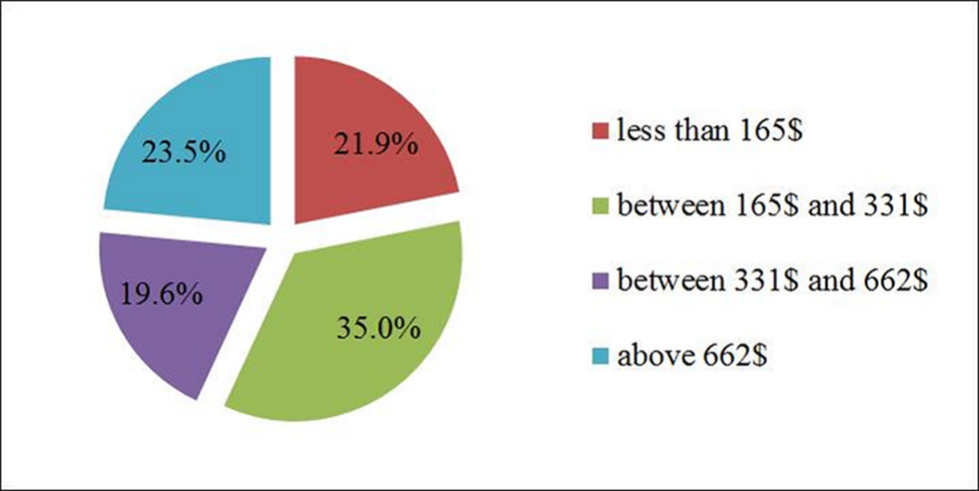
How much money do herdsmen spend on gifts between neighbors in a year?

Most of herdsmen now live in towns, replacing the previous solo living far away from each other. This can establish social norms that promote cohesion, repair and strengthen family cohesion, and provide safe, equitable labor environments that are beneficial to the development of healthy relationships[5].According to Chinese habits, gift exchanges between neighbors at major life events are an indicator of their social relationship. However, 21.9% of the herdsmen’s gift expenditures are below165 $, gift expenditures between 165$and 331$ accounted for the largest proportion (35.0%), while 19.6% had expenditures between 331 $ and 662 $ and 23.5% was able to spend more than 662 $ (Fig 5). Compared with farmers’ yearly gift expenditure of over 496$[44], the average herdsman’s gift expenditure is lower, which may be related to the herdsman’s income.

#### 3.2.7 The right and freedom of choice

**Fig 6:**
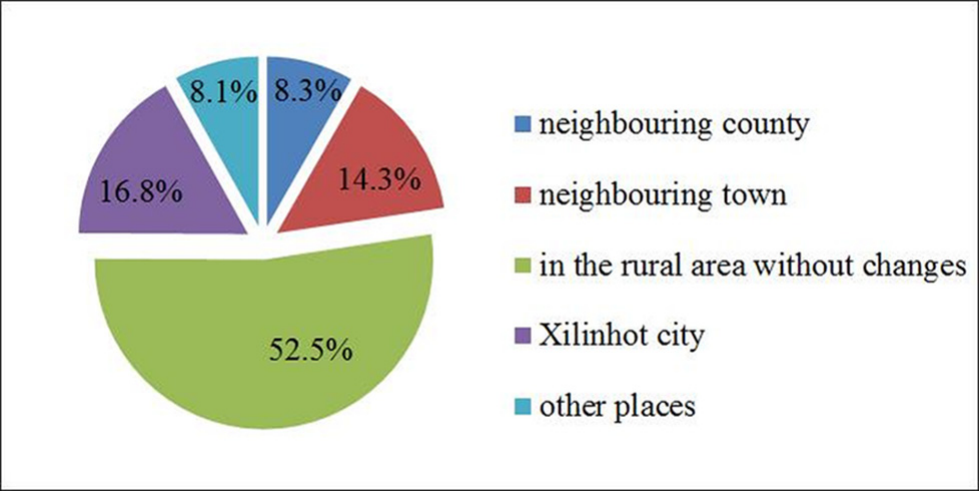
Where do the herdsmen prefer to live?

When asked “where would you like to live?”, 52.5% of herdsmen responded that they like the grassland and prefer to live in rural areas than in the city.They believe that the air is fresher and there is less pollution.On the other hand, 16.8% of the herdsman want to move to Xilinhot,the capital of Xilinguole League, and 30.7% would move to either a neighbouring town or country or elsewhere, because they think that transportation is much more convenient, it is easy for their children to go to school, and they can benefit from more information and opportunities for personal development(Fig 6). However, since more and more grassland is occupied by coal factories, some herdsmen lose the freedom to graze and forage in the grassland.This change directly impacts the emotional wellbeing of herdsmen and gives rise to uncertainty about the future.

**Fig 7:**
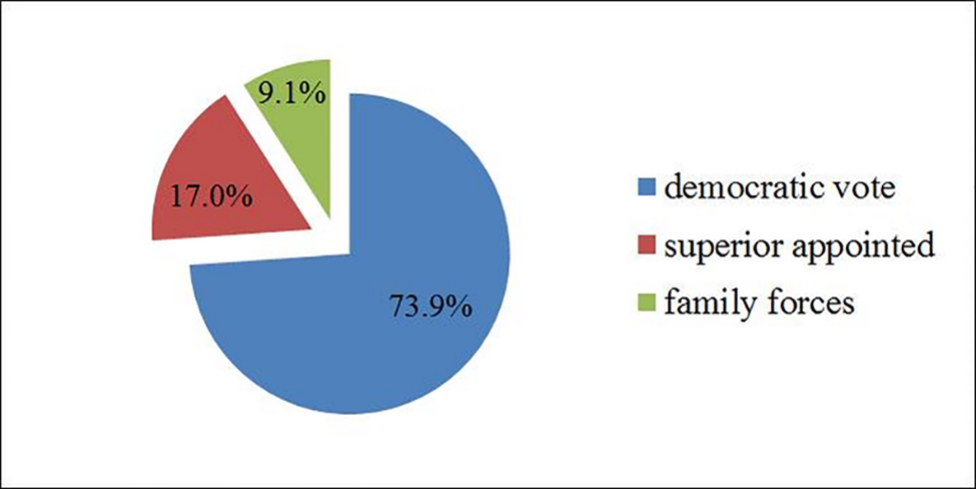
Which are the cadres elected in your village and town?

Regarding democracy rights (e.g., the election of village cadres), 73.9%of the herdsmen reported that the cadres of the towns and villages were selected by their democratic votes, while 9.1%and17.0% of the herdsmen thought that the cadres were appointed respectively by family or by higher authorities. These data indicate that herdsmen perceive to have exercised their democratic rights when selecting local political leaders. The perception that their own values are respected and lead to appoint the desired administration should be considered an improvement of personal herdsmen well-being(Fig 7).

## Discussion

Ecosystem services in the Xilinguole region have undergone important changes under the influence of human activities. These changes are so important that people have recently become more aware of them. Human activities, including excess grazing, over-mining and land reclamation, contribute to environmental pollution and degradation of ecosystem services[45–49]. Changing natural factors (climate change, drought) are also affecting the integrity of local ecosystems, which is becoming increasingly challenging for herdsmen to continue livestock grazing on the grassland.

### 4.1 The influence of economic activities on herdsmen’s wellbeing

As the magnitude of the impact of human activities on ecological systems becomes day-by-day more apparent, there is growing awareness of the link between the ecosystem integrity and human health, economic development, social justice and national security [50].Practices such as intensive grazing, coalmining, tourism and farming in grassland greatly affect human health and wellbeing to different extents.

#### 4.1.1 The influence of overgrazing

Only 30.7% of herdsmen reported that their grassland was seriously overgrazed, 43.2% of herdsmen thought that the amount of their livestock was suited for their grassland area, and 26.1% of herdsmen reported that their grassland area was enough for their livestock to survive (Table 8). They thought the main cause of poor environment is climate change and abnormal weather, and 35.6% thought it resulted from “the waste gas, the waste water, the solid waste” emissions from factories. Only 25.7% of herdsmen thought their overgrazing caused this problem. Overgrazing, when occurring, results into a larger area of degraded grassland [51],and obviously reduces the primary productivity of grassland itself, affects soil nutrients cycling, increases soil erosion and reduces the soil water-holding ability, thus affecting the income of herdsmen. Even small changes to landscapes and grassland ecosystem and the subsequent changes of grazing services could have massive implications for those dependent on animal husbandry[52]. Measures to adjust the herdsmen’s lifestyle and seek alternative grassland development practices with a focus on sustainability are needed[53].

**Table 8:** Herdsmen’s concerns towards pasture overload and environmental degradation?

**Is your pasture overloaded?**		
Yes, it is severely overloaded	No, it is just right for the grassland area	No, the pasture is enough for livestock
30.7%	43.2%	26.1%
**What do you think causes the environmental degradation?**		
Overgrazing	Wastes from factories	Climate change and abnormal weather
25.7%	35.6%	38.7%

#### 4.1.2 The influence of coalmining on herdsmen’s well-being

The increased coal exploitation did not directly change the local herdsmen’s fuel use. Most of them still use cow dung and sheep manure as fuel. The mined coal, which is a nonrenewable resource, is mainly traded and used elsewhere. Only 9.8% of herdsmen reported that they benefited from grassland mining, but 50.1% herdsman reported that the coal mining could enhance their income. Considering that coal exploitation also affects the environment and local quietude, losses seem to outweigh gains. With the presence of coal enterprises, followed by road construction, the establishment of the transit stations and the laying of pipeline, the original and beautiful natural prairie landscape has been replaced by mining landscape, which changes the prairie pattern, damages the culture and quietude of the grasslands and reduces their tourism value[40]. Coal exploitation in grassland is likely to take up a non-negligible fraction of grassland and convert it into waste, while increased noise would harm the herdsmen’s health[54], impact the residents’ quality of life and the stability of social relations and reduce the herdsmen’s well-being.Both positive and negative aspects of mining seem to be perceived by herdsmen and governmental officers; as a consequence, the exploitation of coal in the grasslands should be carried out very carefully and possibly minimized whenever its influence on the grassland ability to provide eco-services becomes evident.

A total of 81.8% of government officials (Table 9) deemed that they should encourage coal exploitation. Two reasons contribute to this: on the one hand, it could increase government revenue, and on the other hand, it can improve the employment rate. However, 18.2% of government officials thought that coal exploitation would destroy the grassland environment and threaten human life reducing people’s wellbeing, so the disadvantages outweigh the advantages. However, approximately 20.0% of government officials deemed that there is a significant security risk in coal mining; approximately 70.0% of government officials thought coal exploitation may produce large amounts of pollutants that would be destructive to the grassland environment, 5.0% of the officials deemed that coal mining would take up considerable grassland area and decrease the grassland for the herdsmen, and 5.0% of government officials thought that coal exploitation would lead to conflict between herdsmen and even impact the development of social relations(Table 9).

**Table 9:** The opinion of government officers concerning the effects of coal exploitation on herdsmens’ well-being.

**What is the most difficult aspect to solve when managing coal mining?**				
Security risks	Polluted grassland environment	Cover grassland area	Disputes between management and herdsmen	
20.0%	70.0%	5.0%	5.0%	
**Should the towns engage in coal mining?**				
It could increase income, so it should be encouraged.	It could increase the employment rate and stability, so it should be encouraged.		It may damage the environment, the disadvantages outweigh the advantages, so it should be opposed.	
54.5%	27.3%		18.2%	

When similar questions were asked to the local herdsmen, only 14.0% deemed that their income increased, 39.3% deemed that their income had not significantly changed, and 46.7% deemed that their income decreased (Table 10). In contrast to the government’s expectations, coal mining in the grassland is only perceived as increasing governmental revenues, notthe herdsmen’s income. The majority (75.8%) of herdsmen believed thatthe coal from the grassland was sold to other places, only 24.2% of herdsmen believed that the exploited coal was used by local herdsmen, and5.6% reported that the mined coal was used as the herdsmen’s fuel for life.

**Table 10:** Advantages and disadvantages of herdsmen from coal exploitation.

**What is the influence of mining on family income?**		
Increased	Changed little	Decreased
14.0%	39.3%	46.7%
**How do coal companies use the mined coal?**		
Meet the daily needs of the local people	Sell the coal to other provinces	
24.2%	75.8%	
**Do you benefit from coal mining?**		
Yes, income increased.	Changed little	Income increased, but the environment was destroyed, the loss out weighs the gain
9.9%	40.0%	50.1%

#### 4.1.3 The influence of tourism development on herdsmen’s well-being

Tourism as an ecosystem service requires the identification of biodiversity, ecosystem and landscape features or assets that drive tourism development as well as of related socio-economic changes[55–56,52]. The future development of the tourism industry is perceived as likely to improve human well-being in the area. The survey showed that 75% of herdsmen thought that their towns had developed a tourism industry, and 15% of pastoralists thought that their township was planning to develop a tourism industry.Moreover,29.1%of herdsmen thought that the travelers in their region increased, 28.4% of herdsmen thought the number of tourists has decreased (Table 11).However, the population of grassland tourists has not changed in recent years.The wide development of grassland tourism promotes the spread of grassland culture, strengthens people’s understanding and promotes cultural exchange between the tourists and pastoralists. However, due to some tourists’, of environmental awareness, some scenic regions are damaged by visitors, and the grassland ecological landscape has been degraded to different extents and even affected the herdsmen’s living environment[40, 57].

**Table 11:** Herdsmen’s opinion about the influence of tourism on their well-being.

**Has your town developed a tourism industry?**		
It has developed one.	It is planning to develop one.	It has no plans to develop.
75%	20%	5%
**Has the amount of visitors changed recently?**		
Increased	Changed little	Decreased
29.1%	42.2%	28.4%

#### 4.1.4 The influence of governmental policies on herdsmen’s wellbeing

Policy decisions related to land use play a significant role in shaping the current state of ecosystems and human well-being in all contexts, thereby implying the need for more informed decision making by governments[58]. The efficacy of the implementation of a policy by the government also depends on the attitude of citizens towards these problems and their understanding of the measures to be implemented under the government’s plan[59].According to 40.3% of the surveyed sample, the governmental policies of “return farmland to forests and animal breeding grounds to grassland” have effectively controlled grassland desertification. Instead, 49.5%of herdsmen thought that this policy had no significant effect on the prevention of desertification, and10.2% thought that desertification was worse than before(Table 12). The herdsmen’s understanding of government policy was however insufficient: only 7.9%of herdsmen declared that they were informed of and understood the government policies’ purpose and significance, while 67.1% of herdsmen knew only a little, and 25.0% of herdsmen did not seem to understand these national policies at all. It is thus clear that the level of the herdsmen’s understanding of governmental policies does not favor their effective implementation. If well understood, eco-compensation policies for sustainable land-use and new practices that diversify the income sources, away from heavy reliance on intensive grazing, could at the same time improve the grassland state and increase the herdsmen well-being[60–61].

**Table 12:** Opinion of herdsmen about the effectiveness of governmental policies for grassland conservation.

**Is governmental policy of turning farmland to grassland effectively preventing desertification?**		
Yes, it effectively controlled it.	Changed little.	No, it is worse than before.
40.3%	49.5%	10.2%
**Do you understand the aim and modalities of governmental grassland policy?**		
Yes, very well.	Yes, but just a little.	No, not at all.
7.9%	67.1%	25.0%

### 4.2 Awareness of links between economic activities and environmental degradation

Several economic and environmental assessments of the grassland ecosystem value to the economy and well-being of local population pointed out and quantified its direct and indirect contribution to the economic development of the region, so that any integrity decrease of the grassland ecosystem is likely to heavily affect the quality of life and the sustainability of the entire local economy and social structure. The survey conducted to test the life standard and environmental awareness of local herdsmen population shows that their life is already affected to some extent by the environmental degradation, but does not show their full awareness of the problems, the responsibilities and the possible solutions. The herdsmen still rely on traditional income patterns (more grassland available, more livestock, more income) which makes most of them hardly able to look out of the box and identify the new options offered by environmental protection and eco-compensation policies for grassland reclamation. Moreover, while coal mining and climate change are recognized among the major factors affecting decreased grassland availability and productivity, the same importance is not assigned to overgrazing due to intensive livestock farming. Changes in the supporting services largely relate to the intensity of grazing. At intensities less than the threshold value, grazing in grassland increases the biomass production and promotes biodiversity.Instead, overgrazing beyond the threshold value restrains grassland primary productivity, destroys the physical behavior of soil and aggravates water loss and soil erosion[38,62]. Thus, the supporting services are affected by human activities and appear to be decreasing.

The grassland regulating services have also decreased under the influence of human factors and natural factors in recent years[63]. The decline in the Xilinguole grassland regulating services results in increased inability to regulate grassland humidity, temperature and pest control, which causes grassland desertification to increase, aggravates erosion, and increases the frequency of natural disasters[29,63,64]. Decreased regulating services also cause loss of livestock production,which could in turn decrease the herdsmen’s income and hinder the improvement of the standard of living.Herdsmen indicated provisioning services as the most important when they were asked about their attitude toward ecosystem services of the grassland. This result reflects the fact that herdsmen pay more attention to the services that can offer direct benefits than those which provide indirect advantages, such as regulating services affecting forage growth or cultural services supporting people’s spiritual lives. The decline of the grassland provisioning services threatens people’s and livestock’s food safety and directly affect the herdsmen’s income, thereby influencing all aspects of basic necessities for life. This may result in a reduction of money for medical expenses and increased risk for people becoming sick, may exacerbate the gap between the rich and the poor, may hinder the development of social relationships and limit herdsmen’s freedom of movement and choice. This is why provisioning services of grassland are perceived and actually are the most important survival and wellbeing factor for herdsmen.

### 4.3 Cultural services

With extensive tourism in grassland areas, the cultural services of grassland have been significantly improved, and herdsmen wellbeing in social relations and health has also obviously been enhanced. Landscape beauty in a well managed grassland attracts tourists from different countries, enhances the cultural exchange between herdsmen and the outside world, promotes the development of other services industries and provides more opportunities for people to choose their occupations. At the same time, it also improves herdsmen’s income and quality of life.

Because of its special geographical location and important ecological functions, the scientific research value of the grassland has also increased. The culture of the grassland attracts a large number of researchers, which makes more people understand the grassland, enhances people’s awareness of the need to conserve its integrity, promotes the implementation of grassland sustainable development strategies and improves human wellbeing.

### 4.4 Environmental protection

Herdsmen are aware of the occurred losses due to environmental degradation. Some herdsmen have also transferred their attention from increasing income to environmental protection and are even willing to invest part of their income to protect the environment and so improve their quality of life. The study of the relationship between ecosystem services and human well-being is very important but also very complex. Clearly, human needs are the driving force behind changes in the ecosystem’s integrity. It is not a given that, once human demand increases beyond the ecosystem carrying capacity, humans stop plundering nature and damaging the ecosystem. A healthy natural environment not only meets the basic human needs but also helps to provide a sense of community by enhancing feelings of pride and a strong sense of kinship among its citizens, who share the common goal of making their community a better place to live [65].

### 4.5 Income and governmental policies

According to the responses to the survey, most herdsmen complained that their income had not significantly changed since 2005. This seems to contradict the data from the statistical office of the Xilinguole League, which report that the average net income of herdsmen was 3,581 RMB in 2005 and 7,471 RMB in 2010, an increase of47.9%. However, rising prices, inflation and the extreme disparity between the rich and the poor may have contributed to that perception, making people feel that their income is steady at the previous level or even decreased. Herdsmen still put basic needs first in terms of well-being and indicate that income dominates their concerns. The herdsmen perceive their income as unchanged or even less than it was before the application of the banning grazing policy. Since 2003, China government started the project of “returning grazing land to grassland”to strictly control and reduce the negative effect of overgrazing. Usually grazing ban mainly was carried out in the high grazing intensity grasslands, but actually it seems very difficult to be implemented since the government owns the grassland that make herdsman care more their animals growth rather than grassland protection. At present, the eco-compensation of grassland is 95.4 RMB(about 15$) per ha in Xilinguole League. The compensation is a way to partially increase income to previous levels, and herdsmen hope that the government will further improve the compensating standard. Based on our survey, government officials believe that the ecological compensation of banning grazing could cover the herdsmen’s losses and that the amount of the compensation is an important factor affecting regional sustainable development. What amount of increase could satisfy herdsmen, how much the government can pay and what type of alternative compensation might be available are topics worth further study and discussion.

## Concluding Remarks

According to the herdsmen questionnaire survey, the main aspects of ecosystem services and human wellbeing changes in grassland are the following: (1) In the recent years, the supporting and provisioning services of the grassland have declined while the culture services of grassland has received more attention than before. Although the referred value of the regulating services is the largest according to literature, in herdsmen’s views the provisioning services were the most important factor of all the ecosystem services influencing human wellbeing. (2) The herdsmen’s cultural exchange, health, education and social relations are better than before, but the threat from different factors to herdsmen’s health and safety increased. (3) The government’s ecological compensation policy did not fully address the losses due to grassland desertification and environmental degradation.The implementation of government policies is still affected by insufficient understanding of their goals by the herdsmen.(4) The most urgent need of the herdsmen is raising their incomes. The herdsmen’s revenue did not increase, but the herdsmen’s exposure to increased survival risk due to grassland degradation and overexploitation did. (5) The influence of grazing, mining and tourism development on the ecosystem services of the grassland depend on the intensity of these activities. Within a certain range, grazing and mining are capable to promote the improvement of the herdsmen’s wellbeing, but once that line is crossed, the upgrading of the herdsman’s living standards is curbed.

## Supporting Information

S1 Appendix The questionnaire of ecosystem services and human well-being change in Xilinguole League. (PDF)
