# Supplementary material for: On the Relationship between Economic Development, Environmental Integrity and Well-Being: The Point of View of Herdsmen in Northern China Grassland
Source: PLoS One. 2015 Sep 2;10(9):e0134786. doi: 10.1371/journal.pone.0134786 (PMC4557951; doi:10.1371/journal.pone.0134786)
Supplement: S1 Appendix — (PDF) [file pone.0134786.s001.pdf]

Appendix

The questionnaire of ecosystem services and human well-being change in Xilinguole League

The background of respondents:

Location: Age: Education level:

Family population: Job: Net income per year:

1. How has the grass grown in recent three years?

A. better B. no significant change C. worse

2. In your view, which grassland ecosystem services are the most important?

A. provisioning services B. regulating services

C. supporting services D. culture services

3. What is the relationship between pasture growth and herdsman life?

A. The pasture grows well, but ht e life is not good.

B. The pasture grows well, and the life is good.

C. The pasture grows bad, but the life is good.

D. The pasture grows bad, and the life is bad.

4. How much is the net annual income of your family?

A. Less than 1,653$

B. Between 1,653$ and 4,959$

C. More than 4,959$

5. How has the net annual income of herdsmen changed?

A. increase B. Change little C. Decrease

6. What caused the income changes?

A. The amount of livestock B. The total yield of grain C. The time of work outside

D. The sum of visitors. E. Other ways

7. Do you have any other income resources besides selling livestock and grain?

A. Yes B. No

8. If the area of grassland is not enough for your needs, what measures should you take?

A. Find part-time work B. Rent more grassland area C. Other

9. Which level represents your living situation?

A. Needy B. Have enough to eat and wear C. Well-off

10. Is there electricity in your house? If there is, how do you obtain it?

A. Not yet B. Yes, coal-fired power plants supply electricity

C. Yes, wind power plants supply electricity

11. Is the area of grassland enough for your grazing needs?

A. Does not meet their needs B. Barely meet their needs C. Fully meet their needs

12. What is the grassland area per person?

A. Less than 66.7 ha B. Between 66.7 ha and 133.4 ha C. More than 133.4 ha

13. What vehicle does your family own?

A. Motorcycle B. Bicycle C. Car D. Truck E. Carriage

14. What is your house made of?

A. Thatched cottage B. Brick house C. Yurt

15. How much money did herdsman spend on seeing a doctor?

A. Less than $165 B. Between $165 and $826

C. Between $826 and $1,653 D. More than $1,653

16. Is there a clinic in your village?

A. Yes B. No

17. Does the village carry an endowment insurance system?

A. Yes B. No

18. Are you satisfied with the endowment insurance system?

A. Yes B. No

19. How have locusts and other diseases changed in the grassland?

A. Aggravate B. No effect C. Weaken

20. Is there enough water for your daily life?

A. Yes, it’s abundant. B. Just meet the need C. No, it’s short of water

21. How do snowstorms affect people’s lives?

A. Heavily effect herdsmen’s normal life B. Small effect, people can live normally

C. No effect

22. Does the snowstorm threat your life safety?

A. Yes, we feel unsafe. B. No, we can control that.

23. What measures have you taken against snowstorms?

A. Purchased or prepare enough forage B. Find a part-time job

C. Ask for help from government D. Take no measures

24. When do the children start to go to school?

A. Before 8 year old B. After 8 year old

25. How far does the nearest primary school from home?

A. 1-3 kilometer B. 3-5 kilometer C. beyond 5 kilometer

26. How far does the nearest middle school from home?

A. Less 5 kilometer B. 5-10kilometer C. Beyond 10 kilometer

27. How does the school facilities?

A. Very good B. Just so so. C. Not good

28. How about the teaching level of school?

A. Very good B. Just so so. C. Not good

29. How much money do herdsmen spend on gifts for neighbors in a year?

A. less than 165$ B. between 165$and 331$

C. between 331$and 662$ D. above 662$

30. Where do the herdsmen prefer to live?

A. Neighbouring county B. Neighbouring town C. Present residence

D. Xilinhot city E. Other places

31. How are the cadres elected in your village and town?

A. democratic vote B. superior appointed C. family forces

32. Is your pasture overloaded?

A. Yes, it is severely overloaded B. No, it is just right for the grassland area

C. No, the pasture is enough for livestock

33. What do you think causes the poor environment?

A. Overgrazing B. Three wastes from factories

C. Climate change and abnormal weather

33. What is the influence of mining on family income?

A. Increased B. Changed little C. Decreased

34. How do they use the mined coal?

A. Meet the daily needs of the local people B. Sell the coal to other provinces

35. Do you benefit from coal mining?

A. Yes, income increased. B. Changed little

C. Income increased, but the environment was destroyed, the loss outweighs the gain

36. What is the most difficult thing to solve when managing coal mining (government officials)?

A. Security risks B. Polluted grassland environment

C. Cover grassland area D. Disputes between management and herdsmen

37. Should the town engage in coal mining (government officials)?

A. It could increase income, so it should be encouraged.

B. It could increase the employment rate and stability, so it should be encouraged.

C. It may damage environment, the disadvantages outweigh the advantages, so it should be opposed.

38. Has your town developed a tourism industry?

A. It has developed one. B. It is planning to develop one.

C. It has no plans to develop one.

39. How has the amount of visitors changed?

A. Increased B. Changed little C. Decreased

40. Do you benefit from tourism development?

A. Yes B. No

41. Is the policy of changing farmland to grassland effectively preventing desertification?

A. Yes, it effectively controlled it. B. Changed little. C. No, it is worse than before.

42. Do you understand the policies of the government?

A. Yes, very well. B. Yes, but just a little C. No, not at all.
